# 1467. Effects Of Oral Antibiotic Prophylaxis For Surgical Site Infections In Colorectal Surgery: Insights From Real World Data

**DOI:** 10.1093/ofid/ofad500.1304

**Published:** 2023-11-27

**Authors:** Hiroaki Hata, Hiroki Nakanishi, Gen Nishikawa, Ryo Matsusue

**Affiliations:** National Hospital Organization Kyoto Medical Center, Kyoto, Kyoto, Japan; National Hospital Organization, Kyoto Medical Center, Kyoto, Kyoto, Japan; National Hospital Organization, Kyoto Medical Center, Kyoto, Kyoto, Japan; National Hospital Organization, Kyoto Medical Center, Kyoto, Kyoto, Japan

## Abstract

**Background:**

Surgical site infection (SSI) after colorectal surgery is a significant burden on healthcare costs and patient quality of life. Preventive strategies are very important to working on this problem. In a multicenter randomized controlled trial (RCT) published in Annals of Surgery(2016;263:1085-1091), we reported the efficacy of oral antimicrobial prophylaxis in preventing SSI before colorectal surgery. Since the end of the RCT in 2012, we have applied this prophylaxis in our clinical practice and have experienced a large number of cases. Here, we report the incidence of SSIs when this prophylactic measure is applied in real-world clinical practice.

**Methods:**

We retrospectively evaluated the incidence of SSI in 1192 patients who underwent elective laparoscopic colorectal resection for colorectal tumors between January 2013 and June 2022. We excluded 24 patients who were unable to receive oral prophylaxis. Antimicrobial prophylaxis consisted of oral kanamycin (1 g) and metronidazole (750 mg) twice on the day before surgery and intravenous cefmetazole (1 g) just before and every 3 hours during surgery. The diagnosis of SSI follows the CDC/NHSN definition.

**Results:**

Our study included 647 males and 545 females, with a mean age of 69.3 years (IQR 63-77) and BMI of 22.6 kg/m^2^ (IQR 20.2-24.7). The incidence of SSI in all surgical cases was 61/1192 (5.1%; 95% CI 4.0-6.5). The incidence of SSIs in colon surgery was 16/769 (2.1%; 95% CI 1.3-3.4), and in rectal surgery, 45/400 (11.3%; 95% CI 8.5-14.7). The incidence of *Clostridioides difficile* colitis, which is regarded as a side effect of oral antimicrobial agents, was low at 6/1192 cases (0.5%; 05%CI 0.2-1.1).

In our previous RCT, the incidence of SSI was 21/289 (7.26%) in patients treated with oral antibiotics. In a subanalysis of this trial published in the International Journal of Colorectal Disease(2016;31:1775-1784), the incidence of SSI was 9/188 (4.8%) in colon surgery and 13/103 (13%) in rectal surgery. The incidence of SSIs in the present data was similar to or lower than the incidence of SSIs in well-conditioned patients selected in the RCT.

**Conclusion:**

Our results suggest that preoperative oral antibiotic prophylaxis in colorectal surgery is an effective method to reduce SSIs in a wide variety of patients in real-world clinical practice.

**Disclosures:**

**All Authors**: No reported disclosures

